# Early childhood general anesthesia exposure associated with later developmental delay: A national population-based cohort study

**DOI:** 10.1371/journal.pone.0238289

**Published:** 2020-09-24

**Authors:** Yu-Pin Feng, Tsorng-Shyang Yang, Chi-Hsiang Chung, Wu-Chien Chien, Chih-Shung Wong

**Affiliations:** 1 Department of Anesthesiology, Cathay General Hospital- Xizhi, New Taipei City, Taiwan; 2 Department of Anesthesiology, Cathay General Hospital, Taipei, Taiwan; 3 School of Public Health, National Defense Medical Center, Taipei, Taiwan; 4 Taiwanese Injury Prevention and Safety Promotion Association (TIPSPA), Taipei, Taiwan; 5 Department of Medical Research, Tri-Service General Hospital, National Defense Medical Center, Taipei, Taiwan; 6 Graduate Institute of Life Science, National Defense Medical Center, Taipei, Taiwan; National Yang-Ming University, TAIWAN

## Abstract

Exposure to general anesthesia has been reported to induce neurotoxicity, impair learning, memory, attention, motor functions, as well as affect behavior in adult rodents and nonhuman primates. Though many have speculated similar effects in humans, previous literature has shown conflicting findings. To investigate the differences in risk of developmental delay among young children exposed to general anesthesia compared to matched unexposed individuals, a population-based cohort study was conducted with a longitudinal dataset spanning 2000 to 2013 from the Taiwan National Health Insurance Research Database (NHIRD). Procedure codes were used to identify children who received anesthesia. For each exposed child, two unexposed children matched by gender and age were enrolled into the comparison cohort. Neurocognitive outcome was measured by the presence of ICD-9-CM codes related to developmental delay (DD). Cox regression models were used to obtain hazard ratios of developing DD after varying levels of anesthesia exposure. After excluding 4,802 individuals who met the exclusion criteria, a total of 11,457 children who received general anesthesia before two years of age was compared to 22,914 children (matched by gender and age) unexposed to anesthesia. Increased risk of DD was observed in the exposure group with a hazard ratio (HR) of 1.320 (95% CI 1.143–1.522, P < 0.001). Subgroup analysis demonstrated further elevated risks of DD with multiple anesthesia exposures (1 anesthesia event: HR 1.145, 95% CI 1.010–1.246, P = 0.04; 2 anesthesia events: HR 1.476, 95% CI 1.155–1.887, P = 0.005; ≥3 anesthesia events: HR 2.222, 95% CI 1.810–2.621, P < 0.001) and longer total anesthesia durations (Total anesthesia <2 hours: HR 1.124, 95% CI 1.003–1.499, P = 0.047; Total anesthesia 2–4 hours: HR 1.450, 95% CI 1.157–1.800, P = 0.004; Total anesthesia > 4 hours: HR 1.598, 95% CI 1.343–1.982, P < 0.001) compared with children unexposed to anesthesia. These results suggest that children exposed to general anesthesia before two years of age have an increased risk of DD. This risk is further elevated with increased frequency of anesthesia, and longer total anesthesia duration. The findings of this study should prompt clinical practitioners to proceed with caution when assessing young patients and planning managements involving procedures requiring general anesthesia.

## Introduction

Recent preclinical evidence demonstrates that drugs for GA may cause structural injury at the cerebral cortex and thalamus as well as long-term neurodevelopment impairment in young animals, including apoptotic cell death and changes in dendritic morphology [1, 2]. These changes have been observed with several different GA agents in studies with rodents and nonhuman primates. Animals of younger age and those exposed to GA agents for longer durations are associated with higher risks [3, 4].

The U.S. Food and Drug Administration (FDA) recently released a new warning regarding the use of anesthetics in children under 3 years of age, raising the awareness to consider postponing pediatric procedures requiring the use of anesthetics until they are older [5]. At the same time, however, delaying necessary procedures may also have unintended harmful consequences [6].

In this retrospective observational study, a cohort of medical insurance enrollees of the Taiwan National Health Insurance (NHI) was used to compare children exposed to GA with matched children with no anesthesia exposure. The purpose of this study is to better understand the association between exposure of different degrees of general anesthesia in pediatric patients and the risks of subsequent developmental disorders.

## Methods

### Ethics

This study was conducted in accordance with the Code of Ethics of the World Medical Association (Declaration of Helsinki). The Institutional Review Board of Tri-Service General Hospital approved this study and waived the need for individual written informed consent (TSGHIRB No. B202005098).

### Study population

The government-run Taiwan National Health Insurance (NHI) enrolls more than 99 percent of the island nation’s population (i.e., more than 23 million people insured). The National Health Insurance Research Database (NHIRD) collects demographic healthcare data with information of clinical visits and hospitalizations, diagnostic codes, prescription profiles, procedures and surgeries, etc. Of all Taiwan’s NHI enrollees, one million patients were randomly selected through stratified probability-sampling method in the year 2000 based on characteristic, such as sex, age, and household income, etc. After eliminating those with incomplete data, a subset of 989,753 patients (with an associated 26,769,418 medical events from January 1, 2000 to December 31, 2013) were selected into the Longitudinal Health Insurance Database (LHID), which is representative of Taiwan’s general population. The database contains inpatient and outpatient diagnostic and treatment codes, health status information, and prescribed medications. Claims are listed chronologically, providing temporal sequence of medical events.

### Assignment of exposed vs. non-exposed cohorts

Different procedures and types of anesthesia (e.g. general, spinal, epidural, nerve block, etc.) along with the duration of anesthesia, are coded differently in the NHI database. In this study, children found to have a code for GA exposure before 2 years of age were assigned to the exposed group or GA cohort. Each child in the exposed group was then matched without replacement (based on gender and age) to two children who were not exposed to GA before 2 years of age. Under ideal conditions, a 4-to-1 comparison-to-study ratio would be optimal for achieving higher statistical power. A 2-to-1 comparison-to-study ratio was used in our study as higher ratios were limited by the number of pediatric patients in the LHID database [7]. With this limitation however, it is still worthwhile to double the number of controls from a 1-to-1 to a 2-to-1 comparison-to-study ratio [8]. These non-exposed children were assigned to the comparison group or non-GA cohort. For the individuals exposed to GA, the observation period was initiated from the day of their first GA exposure. The matched non-exposed individuals were enrolled on the same day as their exposed counterparts. All study subjects were continuously observed until codes of International Classification of Diseases, Clinical Modification (ICD-9-CM) of developmental delays was registered on the database. Developmental delay ICD-9-CM codes include 299, 312.81, 312.89, 312.9, 313–315, 317–319, 783.42, V79.8, and V79.9 [9]. Observation of all remaining individuals without a developmental delay (DD) diagnosis ended on December 31, 2013, the last day of data collection of the LHID. Due to the different times of enrollment for every set of matched exposed-unexposed children, the observation period is different for every individual.

### Data processing

The duration of anesthesia in individuals of the exposed group were identified by the LHID codes used in the database. Specifically, GA with endotracheal tube was coded as 96020C (< 2hr), 96021C (2hr-4hr), 96022C (> 4hr), while GA with laryngeal mask airway was coded as 96017C (< 2hr), 96018C (2hr-4hr), and 96019C (> 4hr).

### Statistical analysis

All analyses were performed using SPSS software version 22 (SPSS Inc., Chicago, Ill., USA). The Fisher exact test was used for categorical variables of case numbers less than five, while Chi-squared test was used for categorical variables of case numbers more than five, to test for differences between exposed and unexposed participants in the cohort at baseline. Categorical variables such as brain cancer, shock, leukemia, heart failure, stroke, and lung contusion were tested with Fisher exact test. To examine the association between general anesthesia and DD, the patients were classified into three subgroups by frequency of anesthesia (once, twice, three or more times), and three subgroups by total duration of anesthesia (less than 2 hours, 2 to 4 hours, more than 4 hours), with summed durations for patients with multiple GA exposures. Four models of multivariate cox proportional hazards regression analyses were used to determine the risk of DD, specifically to examine the impacts of anesthesia exposure, anesthesia frequency, total anesthesia duration, and the combined effects of frequency and total duration of anesthesia. The results are presented as hazard ratios (HR) with 95% confidence intervals (CI). The difference in the risk of DD between the GA and non-GA cohorts was estimated using the Kaplan–Meier method with the log-rank test. A two-tailed p-value of less than 0.05 indicates statistical significance.

## Results

**Fig 1:**
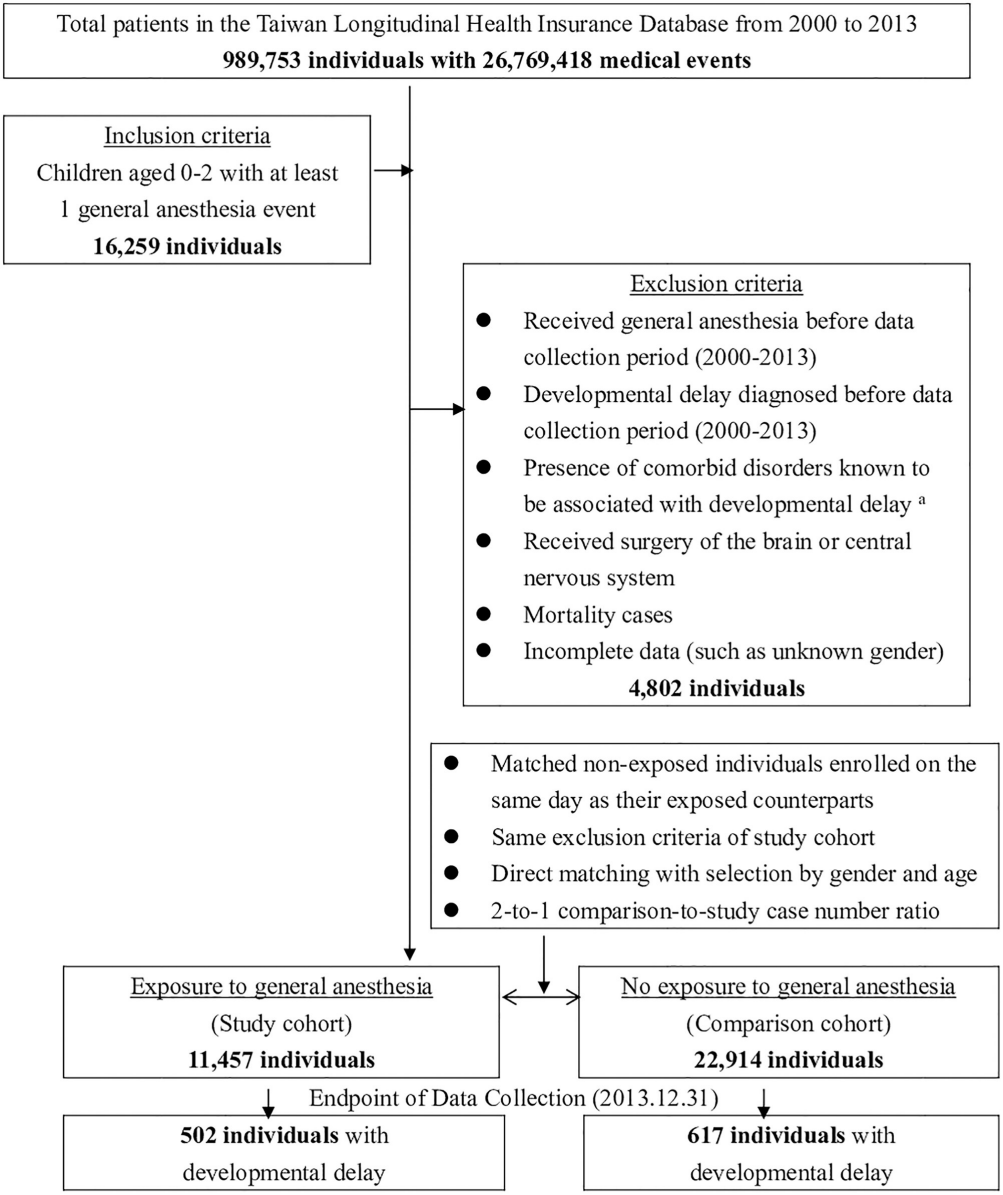
Algorithm of the study design and patient selection. ^a^ Developmental delay related disease: ICD-9-CM 243 (Congenital hypothyroidism), 250–259 (Diabetes mellitus), 320–326 (Inflammatory diseases of the central nervous system), 330–331 & 333–337 (Hereditary and degenerative diseases of the central nervous system), 343–345 (Cerebral palsy, paralytic syndromes, epilepsy), 740–744 (Congenital anomalies of the head and nervous system), 758–759 (Chromosomal anomalies), 765 (Disorders relating to short gestation and low birth-weight), 768–771 (Disorders relating to distress and infections in perinatal period), 775 (Endocrine and metabolic disturbances specific to the fetus and newborn).

Of the 989,753 individuals in the LHID from 2000–2013, 16,259 were identified as having been exposed to general anesthesia (either with endotracheal tube or laryngeal mask) before 2 years of age. Of this group, 4,802 patients were then excluded, including those already diagnosed with developmental delay, those who received neurosurgeries, mortality cases and incomplete data entries (such as unknown gender), with a remaining total of 11,457 patients assigned to the GA cohort as the study group. Another 22,914 children were matched for age and gender, assigned to the non-GA cohort as the comparison group using the same exclusion criteria as the study group. A total of 34,371 children were examined in this study, with the selection algorithm presented in Fig 1.

The demographic characteristics of patients included in this study are listed in Table 1, grouped by gender, household income, catastrophic illness certificate status, mean age at time of enrollment, comorbidities, level of facility where medical care was received, and length of hospitalization. Low income households in Taiwan are defined as those with a monthly average per-member gross income of less than the monthly minimum living expense standard in the household’s residence region [10]. The GA cohort comprised of a higher ratio of low-income households compared to the non-GA cohort (2.47% versus 2.00%, P = 0.005). Enrollees of the National Healthcare Insurance (NHI) with diagnoses of disabling diseases are issued catastrophic illness certificates, with the benefit of subsidized medical bills. The GA cohort consists of a higher proportion of catastrophic illness certificate holders compared to the non-GA cohort (0.62% versus 0.43%, P = 0.022). To account for comorbidities, the pediatric comorbidity index was used to reflect the age group of our study subjects [11, 12]. Comorbidities were included as binary variables (either present or absent) according the ICD-9 codes that have been assigned for both exposed and unexposed patients at the end of observation (either the event of DD diagnosis or termination of LHID data collection on December 31, 2013). Comorbidities were compared between the exposed and unexposed cohorts, with no significant differences found apart from a higher frequency of pneumonia patients in the GA cohort compared to the non-GA cohort (0.58% versus 0.41%, P = 0.027). The GA cohort tended to receive medical care in larger medical centers (73.8%) rather than in medium-sized regional or smaller local hospitals (p < 0.001). For the study cohort exposed to GA, length of hospital stay is defined as the total number of days hospitalized for surgery divided by the number of admissions for surgeries. For the comparison non-exposed cohort, length of hospital stay is defined as the total number of days hospitalized divided by the number of hospital admissions. The GA cohort showed longer hospitalizations compared to the non-GA cohort (4.45 days versus 4.23 days, P < 0.001).

**Table 1:** Demographic characteristics of the general anesthesia (GA) cohort versus the comparison (non-GA) matched cohort.

		non-GA matched (*n* = 22,914)	GA cohort (*n* = 11,457)	P-value
Male [*n* (%)]		13,362 (58.31)	6,681 (58.31)	0.999
Low-income households [*n* (%)]		458 (2.00)	283 (2.47)	0.005
Catastrophic illness certificate holders [*n* (%)]		99 (0.43)	71 (0.62)	0.022
Mean age at enrollment [years old (SD)]		0.78 ± 0.60	0.77 ± 0.59	0.143
Age at enrollment (months)	≦6 months [*n* (%)]	9,208 (40.19)	4,604 (40.19)	0.999
	>6 months, ≦12 months [*n* (%)]	6,342 (27.68)	3,171 (27.68)	
	>12 months, ≦18 months [*n* (%)]	4,412 (19.25)	2,206 (19.25)	
	>18 months, ≦24 months [*n* (%)]	2,952 (12.88)	1,476 (12.88)	
Pediatric Comorbidities	Brain cancer [*n* (%)]	2 (0.01)	0 (0.00)	0.555
	Diabetes insipidus [*n* (%)]	0 (0.00)	0 (0.00)	N/A
	Asphyxia [*n* (%)]	5 (0.02)	4 (0.03)	0.494
	Shock [*n* (%)]	0 (0.00)	2 (0.02)	0.112
	Leukemia [*n* (%)]	2 (0.01)	4 (0.03)	0.101
	Heart failure [*n* (%)]	3 (0.01)	2 (0.02)	0.757
	Feeding problem [*n* (%)]	0 (0.00)	0 (0.00)	N/A
	Pneumonitis [*n* (%)]	0 (0.00)	1 (0.01)	0.335
	Stroke [*n* (%)]	3 (0.01)	1 (0.01)	0.720
	Candidiasis [*n* (%)]	0 (0.00)	1 (0.01)	0.335
	Head injury [*n* (%)]	7 (0.03)	4 (0.03)	0.838
	Acidosis [*n* (%)]	0 (0.00)	0 (0.00)	N/A
	Hypertension [*n* (%)]	0 (0.00)	0 (0.00)	N/A
	Respiratory failure [*n* (%)]	21 (0.09)	11 (0.10)	0.913
	Lung contusion [*n* (%)]	0 (0.00)	1 (0.01)	0.335
	Ventricular septal defect [*n* (%)]	6 (0.03)	3 (0.03)	0.999
	Congenital subaortic stenosis [*n* (%)]	0 (0.00)	0 (0.00)	N/A
	Arrhythmia [*n* (%)]	254 (1.11)	151 (1.32)	0.109
	Septicemia [*n* (%)]	39 (0.17)	18 (0.16)	0.882
	Coagulopathy [*n* (%)	0 (0.00)	1 (0.01)	0.335
	Agranulocytosis [*n* (%)]	0 (0.00)	1 (0.01)	0.335
	Pyrexia [*n* (%)]	0 (0.00)	0 (0.00)	N/A
	Hydrocephalus [*n* (%)]	1 (0.00)	2 (0.02)	0.261
	Pneumonia [*n* (%)]	93 (0.41)	67 (0.58)	0.027
	Femur fracture [*n* (%)]	2 (0.01)	1 (0.01)	0.996
	Seizure [*n* (%)]	18 (0.08)	12 (0.10)	0.444
	Preterm labor and small for gestational age [*n* (%)]	0 (0.00)	0 (0.00)	N/A
	Perinatal complications [*n* (%)]	5 (0.02)	4 (0.03)	0.494
	Autistic spectrum disorder [*n* (%)]	0 (0.00)	1 (0.01)	0.335
	Intellectual disability [*n* (%)]	0 (0.00)	1 (0.01)	0.335
	Other disorders of the central nervous system [*n* (%)]	3 (0.01)	2 (0.02)	0.757
	Infantile cerebral palsy and epilepsy [*n* (%)]	0 (0.00)	1 (0.01)	0.335
	Attention deficit hyperactivity disorder [*n* (%)]	0 (0.00)	1 (0.01)	0.335
	Otitis media [*n* (%)]	0 (0.00)	0 (0.00)	N/A
	Hearing loss [*n* (%)]	0 (0.00)	0 (0.00)	N/A
Level of care facility	Medical center [*n* (%)]	6,825 (29.79)	8,455 (73.80)	<0.001
	Regional hospital [*n* (%)]	8,998 (39.27)	2,933 (25.60)	
	Local hospital [*n* (%)]	7,091 (30.95)	69 (0.60)	
Length of hospital stay (days) [mean (SD)]		4.23 (3.10)	4.45 (4.21)	<0.001

Note: P-values are derived with Chi-square/Fisher exact test on category variables and t-test on continuous variables.

To account for covariates which may confound or mask associations, multivariate analyses using Cox proportional hazard regression models was performed as shown in Table 2, stratifying for exposure for GA, total duration of GA, frequency of GA, and adjusting for covariates as listed in the pediatric comorbidity index. In the Cox proportional hazard regression model 1, children exposed to anesthesia had a significantly higher risk of a DD diagnosis compared to those without (hazard ratio 1.320, 95% confidence interval [CI] 1.143–1.522, P < 0.001). In model 2, hazard ratio for DD diagnosis increased from 1.124 to 1.598 with longer total anesthesia duration. In model 3, hazard ratio also increased from 1.145 to 2.222 with increased frequency of exposures.

**Table 2:** Comparing hazard ratios of developmental delay using multivariate cox regression in four models with varying levels of general anesthesia (GA) exposure.

		Model 1: Anesthesia exposure				Model 2: Total anesthesia duration				Model 3: Anesthesia frequency				Model 4: Total duration & frequency of anesthesia			
		Adjusted HR	95% CI	95% CI	P-value	Adjusted HR	95% CI	95% CI	P-value	Adjusted HR	95% CI	95% CI	P-value	Adjusted HR	95% CI	95% CI	P-value
Anesthesia exposure		1.320	1.143	1.522	<0.001												
Total anesthesia duration	<2 hrs					1.124	1.003	1.499	0.047					1.813	1.345	2.444	<0.001
	2–4 hrs					1.450	1.157	1.800	0.004					2.044	1.772	2.965	<0.001
	>4 hrs					1.598	1.343	1.982	<0.001					2.197	1.987	3.119	<0.001
Anesthesia frequency	1									1.145	1.010	1.246	0.040	1.520	1.033	1.896	0.037
	2									1.476	1.155	1.887	0.005	1.742	1.569	1.931	<0.001
	≧3									2.222	1.810	2.621	<0.001	1.798	1.601	2.114	<0.001
Male		1.458	1.253	1.701	<0.001	1.424	1.204	1.862	<0.001	1.460	1.333	1.897	<0.001	1.473	1.385	1.979	<0.001
Low-income households		0.481	0.262	1.876	0.172	0.387	0.268	1.985	0.199	0.432	0.310	1.900	0.257	0.469	0.392	1.278	0.380
Catastrophic illness certificate status		6.948	4.593	10.515	<0.001	6.450	4.226	10.750	<0.001	6.497	4.295	9.795	<0.001	6.308	4.001	10.013	<0.001
Seizure		2.245	1.269	3.968	0.005	2.211	1.234	3.897	0.001	2.241	1.220	3.880	0.008	2.495	1.342	4.915	<0.001
Age of initial GA exposure (months)	≦6 months	Reference				Reference				Reference				Reference			
	>6 to ≦12 months	0.940	0.781	1.111	0.481	0.934	0.811	1.222	0.398	0.984	0.713	1.299	0.480	0.995	0.810	1.201	0.624
	>12 to ≦18 months	0.768	0.630	0.938	0.010	0.756	0.531	0.934	0.025	0.767	0.645	1.004	0.058	0.835	0.613	1.049	0.148
	>18 to ≦24 months	0.410	0.304	0.551	<0.001	0.305	0.297	0.663	<0.001	0.565	0.403	0.682	<0.001	0.551	0.392	0.795	<0.001
Level of care facility	Medical center	5.448	2.471	7.184	<0.001	5.466	2.276	7.311	<0.001	5.518	2.719	7.390	<0.001	5.542	2.824	7.721	<0.001
	Regional hospital	3.032	1.570	4.158	<0.001	3.143	1.680	4.390	<0.001	3.225	1.794	4.942	<0.001	3.366	1.897	5.140	<0.001
	Local hospital	Reference				Reference				Reference				Reference			
Length of hospital stay		1.064	0.827	1.412	0.359	1.064	0.822	1.407	0.417	1.065	0.796	1.475	0.404	1.153	0.740	1.762	0.473

HR = Hazard ratios, CI = Confidence Intervals, Adjusted HR: Hazard ratios after adjusting for sex, Low-income households, Catastrophic illness certificate status, Age of initial GA exposure, Level of care facility, Level of care facility, Length of hospital stay, and comorbidities including Brain cancer, Diabetes insipidus, Asphyxia, Shock, Leukemia, Heart failure, Feeding problem, Pneumonitis, Stroke, Candidiasis, Head injury, Acidosis, Hypertension, Respiratory failure, Lung contusion, Ventricular septal defect, Congenital subaortic stenosis, Arrhythmia, Septicemia, Coagulopathy, Agranulocytosis, Pyrexia, Hydrocephalus, Pneumonia, Femur fracture, Seizure, Preterm labor and small for gestational age, Perinatal complications, Autistic spectrum disorder, Intellectual disability, Other disorders of the central nervous system, Infantile cerebral palsy and epilepsy, Attention deficit hyperactivity disorder, Otitis media, Hearing loss. Note: After adjusting for covariates using multivariate cox regression, the risk for developing DD was significantly higher in the GA cohort compared to the non-GA cohort, especially when exposure to GA occurred before reaching 1 year of age. Seizure was the only comorbidity with an increased adjusted hazard ratio reaching statistical significance.

In Model 1, boys showed a higher risk of DD than girls (hazard ratio 1.458, 95% CI, 1.253–1.701, P <0.001). Low-income households did not show statistical significance after adjusting for covariates under Cox’s model. Those issued with catastrophic illness certificate had a significantly higher risk of DD (hazard ratio 6.948, 95% CI, 4.593–10.515, P <0.001). The diagnosis of seizure also showed higher risk of DD (hazard ratio 2.245, 95% CI, 1.269–3.968, P <0.001).

Pediatric patients whose age of initial exposure to anesthesia was 12–18 months old showed reduced risk for DD (hazard ratio 0.768, P = 0.010) compared to those who were exposed to anesthesia before 6 months old. This risk of DD is further reduced (hazard ratio 0.410, P <0.001) in patients first exposed to anesthesia at 18–24 months old compared to those exposed before 6 months old. Risk of DD was higher when receiving medical care in larger medical centers compared to smaller local hospitals (hazard ratio 5.448, P <0.001). Similarly, risk of DD was higher when receiving medical care in medium-sized regional hospitals compared to smaller local hospitals (hazard ratio 3.032, P <0.001).

**Fig 2:**
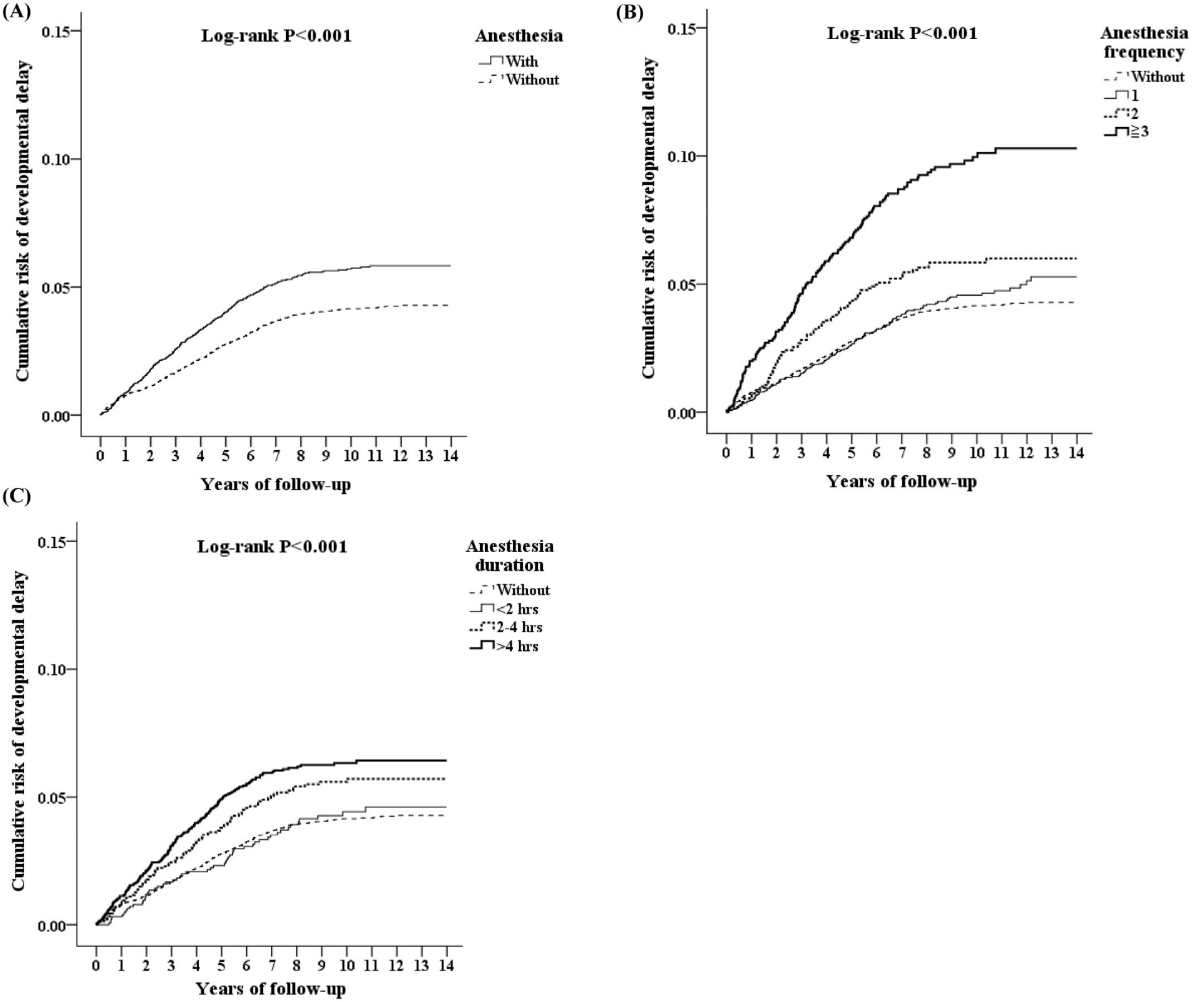
Kaplan-Meier curves for the cumulative risks of developmental delay in children before the age of two. (A) Children who were exposed to GA under the age of two, showed higher risk of DD than those who did not. (B, C) Among children who received GA under the age of two, the risk of DD was increased with frequency and total duration of GA. These analyses were stratified using the log-rank test (p < 0.001).

Cumulative risks of developmental delay in children after GA were computed into Kaplan-Meier curves as seen in Fig 2. The difference in cumulative risk of DD becomes evident with time in patients exposed to anesthesia compared to those without. These cumulative risks were further increased with more frequent anesthesia exposure and longer total anesthesia duration. These analyses were stratified using the log-rank test (P < 0.001). It should be noted that the KM curves only account for age and gender of the patients, but not the remaining covariates (catastrophic illness, seizure, level of care facility, etc).

**Fig 3:**
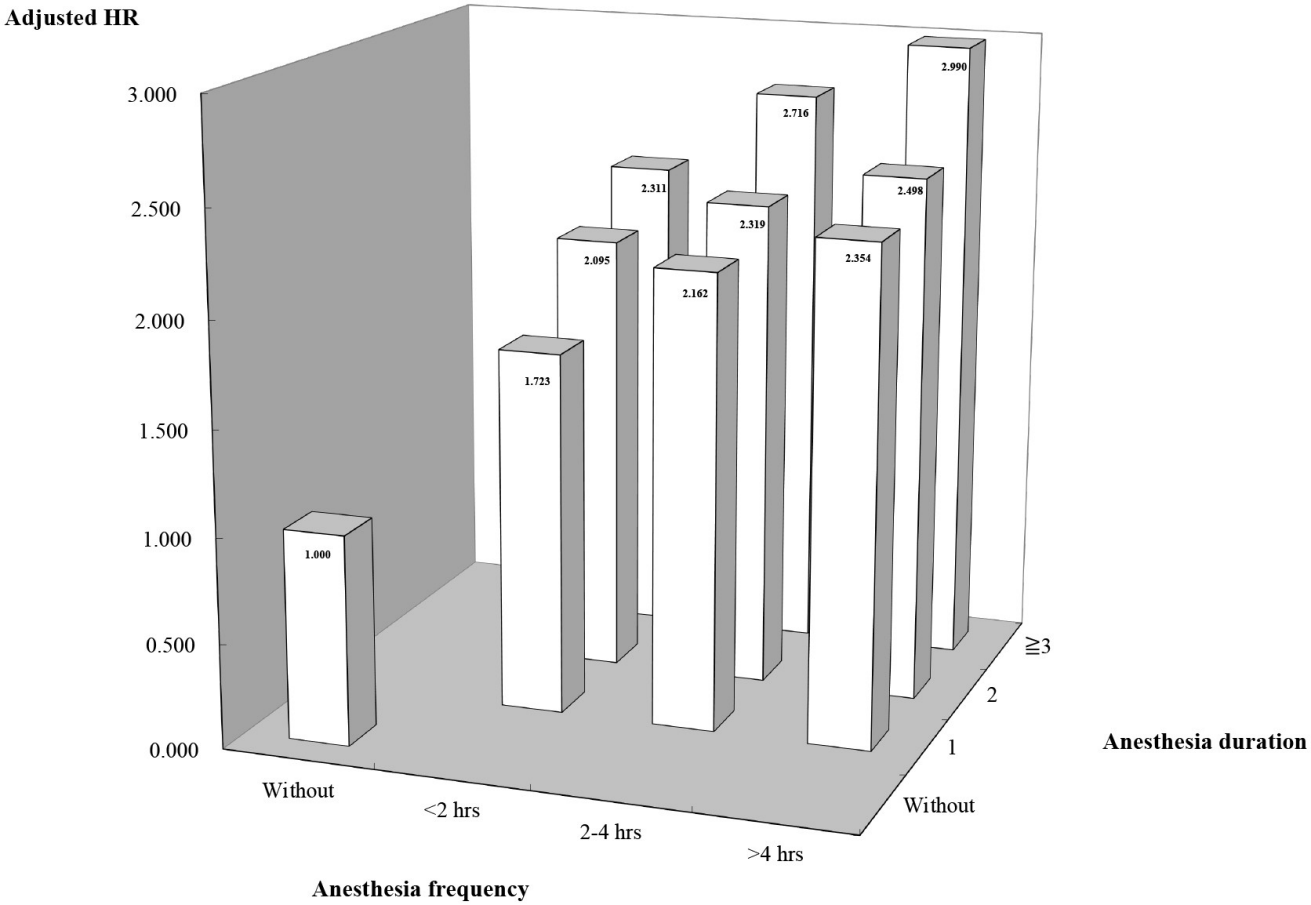
Dose-response 3D plots for the effect of total duration (x axis) and frequency (y axis) of general anesthesia (GA) on the adjusted hazard ratios (HR) (z axis) of developmental delay (DD). Covariates (Brain cancer, Diabetes insipidus, Asphyxia, Shock, Leukemia, Heart failure, Feeding problem, Pneumonitis, Stroke, Candidiasis, Head injury, Acidosis, Hypertension, Respiratory failure, Lung contusion, Ventricular septal defect, Congenital subaortic stenosis, Arrhythmia, Septicemia, Coagulopathy, Agranulocytosis, Pyrexia, Hydrocephalus, Pneumonia, Femur fracture, and Seizure) have been accounted for, adjusting with multivariate Cox regression.

The combined effects of anesthesia frequency and total anesthesia duration on risk of DD can be visualized with the dose-response 3D plot graph as presented in Fig 3. The hazard ratios were adjusted for gender, household income, catastrophic illness certificate status, age of initial exposure to anesthesia, comorbidities, level of facility where medical care was received, and length of hospitalization. More frequent anesthesia exposure and longer total anesthesia duration and showed increasingly higher risks of subsequent DD.

Within the GA cohort in this study, the different surgeries received by the children are listed in Table 3. The most common surgeries requiring general anesthesia in this age group are dental treatment (45.42%), general surgeries (43.46%), urological surgeries (8.82%), and ophthalmological surgeries (0.01%).

**Table 3:** The most common surgeries received by the GA cohort (n = 11,457).

Type of treatment or surgery	Number of patients	% of patients
Dental treatment	5205	45.42
General surgery	4979	43.46
Urology	1010	8.82
Ophthalmology	121	0.01
Other	141	0.01

The comparison of GA exposure on subsequent DD risks on patients receiving different types of surgeries is shown in Table 4. The increased adjusted hazard ratio of subsequent DD is statistically significant in the GA cohort compared to the non-GA cohort in patients receiving general and urological surgery. This statistical significance of increased DD risk after GA exposure is not observed in patients receiving dental surgery.

**Table 4:** Comparison of risk of developmental delay according to surgical type (n = 11,457).

	GA cohort		non-GA matched		GA cohort vs. non-GA matched
	Total *(n)*	DD *(n)*	Total *(n)*	DD *(n)*	Adjusted HR (95% CI), *P* value
Dental treatment	5,205	18	10,986	30	1.027 (0.890–1.185), *P* = 0.243
General surgery	4,979	325	1,025	18	3.105 (2.611–3.477), *P* < 0.001
Urology	1,010	140	1,114	57	2.198 (1.903–2.534), *P* < 0.001
Others	263	19	9,789	512	1.120 (0.970–1.292), *P* = 0.072
**Overall**	11,457	502	22,914	617	1.320 (1.143–1.522), *P* < 0.001

## Discussion

The results of this population-based cohort study reveal that children exposed to GA are at higher risks of subsequent DD (Table 5). The hazard ratio of DD was calculated to be 1.320 in the exposure group compared to the non-exposure group during the observation period from 2000 to 2013 (Table 2, Model 1). This association between GA and DD is compatible with animal findings, which may also be the result of combined effects of surgical stress, the underlying disease pathology, or other comorbidities of the developing brain. Though previous studies claimed that single exposures to anesthetic did not affect neurodevelopment [13, 14], recent studies have found that even short anesthetic exposures (<2 hours) may have detrimental long-term effect on the neurodevelopment in children [15–17].

**Table 5:** Comparing incidences of developmental delay with varying levels of general anesthesia (GA) exposure.

		Non-GA matched (*n* = 22,914)	GA cohort by total duration (*n* = 11,457)			P-value	GA cohort by frequency (*n* = 11,457)			P-value
			<2 hrs (*n* = 2,449)	2–4 hrs (*n* = 3,494)	>4 hrs (*n* = 5,514)		1 (*n* = 5,881)	2 (*n* = 2,873)	≧3 (*n* = 2,703)	
Developmental Delay [n (%)]		617 (2.69)	69 (2.82)	108 (3.09)	325 (5.89)	>0.05	198 (3.37)	156 (5.43)	148 (5.48)	>0.05
			502 (4.38)			<0.001	502 (4.38)			<0.001
Gender	Male [*n* (%)]	13,362 (58.31)	1,440 (58.80)	2,081 (59.56)	3,160 (57.31)	0.190	3,255 (55.35)	1,689 (58.79)	1,737 (64.26)	<0.001
	Female [*n* (%)]	9,552 (41.69)	1,009 (41.20)	1,413 (40.44)	2,354 (42.69)		26,26 (44.65)	1,184 (41.21)	966 (35.74)	
Low-income households [*n* (%)]		458 (2.00)	76 (3.10)	72 (2.06)	135 (2.45)	0.001	137 (2.33)	55 (1.91)	91 (3.37)	<0.001
Catastrophic illness certificate holders [*n* (%)]		99 (0.43)	10 (0.41)	10 (0.29)	51 (0.92)	<0.001	25 (0.43)	17 (0.59)	29 (1.07)	<0.001
Level of care facility	Medical center [*n* (%)]	6,825 (29.79)	1,865 (76.15)	2,615 (74.84)	3,975 (72.09)	<0.001	4,251 (72.28)	2,125 (73.96)	2,079 (76.91)	<0.001
	Regional hospital [*n* (%)]	8,998 (39.27)	564 (23.03)	868 (24.84)	1,501 (27.22)		1,612 (27.41)	711 (24.75)	610 (22.57)	
	Local hospital [*n* (%)]	7,091 (30.95)	20 (0.82)	11 (0.31)	38 (0.69)		18 (0.31)	37 (1.29)	14 (0.52)	
Length of hospital stay (days) [mean (SD)]		4.23 (3.10)	4.01 (3.52)	4.27 (3.89)	4.75 (4.63)	<0.001	4.16 (3.65)	4.64 (4.85)	5.08 (4.52)	<0.001

Note: P-values are derived with Chi-square/Fisher exact test on category variables and One-way ANOVA with Scheffe post hoc on continuous variables

Younger age at initial exposure to GA was identified as a risk factor for developing DD, especially in those exposed to anesthesia prior to 1 year of age, even after adjusting for underlying patient comorbidities and other covariates. This corresponds with neurotoxicity studies on rats [1, 2, 4]. Neuron proliferation in the brain has been found to occur more rapidly in early development [18], and GA may trigger apoptosis during neuronal development. Previous publications on this topic however, have found mixed results [3, 17, 19–21]. Even though a sibling-matched cohort study of 105 sibling pairs receiving anesthesia before 36 months old found no statistical difference in IQ scores after 8–15 years of follow-up [22], other population-based cohort studies suggested that children exposed to GA in early life may have a higher risk of lower academic performance [16, 23]. However, these studies were limited in their ability to define the relationship between multiple exposures and increased duration of anesthesia with subsequent neurodevelopmental risks. One cohort study revealed that the frequency of multiple operations was associated with an increased risk of developmental disability, but exposure of anesthesia was indirectly deduced due to the fact that anesthesia method was not a recorded item in their database [15].

Multiple and extended exposures to GA were identified as risk factors for development delay in this study. The most common diagnosis for children in this age group requiring multiple and extended general anesthesia was found to be complicated hernias. Recent studies of the gut-brain axis suggest that the gut microbiome may play a role in neurodevelopment. Further research will be needed to determine whether or not complicated hernias itself or the process of treating complicated hernias affects the brain-gut axis in young developing brains [24, 25]. Studies have shown that children who received multiple procedures are more likely to develop significant conditions or chronic diseases compared to those who did not receive multiple procedures [26].

Not surprisingly, patients issued with the catastrophic illness certificate showed higher risks of developing DD [26, 27]. This could be due to the patients being debilitated by underlying diseases and illnesses, thus unable to participate in regular daily activities and physical stimulations.

Development delay in boys is well documented [28]. The results of this study also revealed a higher hazard ratio of DD of 1.46 for boys compared to girls (95% CI 1.253–1.700, P <0.001). Some have proposed a genetic influence, with X-linked genetic diseases in boys contributing to the increased incidences of neurodevelopmental disorders [29].

With or without GA, several studies have found that children with epilepsy suffered worse language performances, especially in those with poor seizure control [30, 31]. The results of this study also showed increased risks for DD in children with seizures with hazard ratios ranging from 2.211 to 2.497 according to different levels of anesthesia exposure (P< 0.05) (Table 2).

We present a population-based retrospective cohort study seeking to identify correlations and trends in pediatric patients. Even though the results of this study propose an association between GA and DD, prospective clinical trials are still the gold standard for conclusive clinical evidence. However, randomizing children to receive anesthesia and procedures at certain ages faces strong ethical and logistical constraints. Thus, large population-based observational study designs may be the most effective method to answer questions related to anesthesia in children. As with any other observational study, there will be always be residual confounding factors which may not be completely accounted for, which should be considered when interpreting results.

A number of limitations were encountered in this study. First of all, among the procedures requiring anesthesia received by the pediatric population, the majority were shown to be hernia and dental-related. This is a result of neurosurgical cases being excluded as part of the exclusion criteria for this study, and many less-invasive procedures performed under intravenous and local anesthesia were not included in the patient selection. However, for uncooperative children younger than the age of two requiring dental treatment, almost all received treatment under general anesthesia with placement of an endotracheal tube for airway protection. The high proportion of dental procedures in this age group may be attributed to Taiwan’s relatively short history of economic growth, with large wealth gaps between urban families and those in the countryside. Many children in rural areas are raised by their grandparents, while their parents work in the cities. This social situation combined with the absence of water fluoridation programs contributes to a high prevalence of severe dental caries in early childhood. Secondly, it is difficult to differentiate the effects of the anesthesia from those of the surgery or any other perioperative complications. Thirdly, detection of neurodevelopmental disorders relied on the correct diagnosis and coding into the administrative database. While there may be misclassifications with the ICD-9 codes concerning whether or not developmental delay is present, due to the independent nature between exposure and disease, non-differential misclassification is preferred, consequently leading to bias toward the null. This would suggest an underestimation of our results compared to the actual association between exposure and disease [32]. Fourthly, the vast variability of general anesthesia provided makes it difficult to determine the impacts of specific types or combinations of anesthetic agents used and their dosage details. These limitations inherently hinder the capacity for cohort studies to conclusively determine the link between anesthesia exposures and neurodevelopment outcomes. Due to the absence of a pediatric comorbidity index specifically designed for developmental delay, the index used for this study was based on comorbidity lists published by Tai D et al. and Tai YM et al. [11, 12]; It should be noted that these were original designed for the prediction of mortality and ADHD in the pediatric population.

Additional information is required on the specific drugs, dosages and durations of anesthesia to determine which general anesthetic agents and/or practices carry greater risks than others, and whether lower risk alternatives are available. Meanwhile, potential neuroprotection strategies to reduce neurotoxicity are being explored [33, 34]. Postponing elective procedures until the children are older has also been recommended [35], pending further study results.

In conclusion, this population-based retrospective cohort analysis shows a positive correlation with exposure of GA and risk of DD in pediatric patients. The large sample size and the longitudinal observation with limited loss to follow-up are the strengths of this study. The Taiwan NHIRD offers comprehensive data on out-patient visits, hospital admissions, prescriptions, diseases, and health status for 99% of the population of Taiwan of 23 million. While further research is needed on this topic, the findings of this study should prompt clinical practitioners to proceed with caution when assessing young patients and planning managements involving procedures requiring general anesthesia.

## Supporting information

S1 File Summary of figures and the statistical methods used. (DOC)
