# Supplementary material for: Early childhood general anesthesia exposure associated with later developmental delay: A national population-based cohort study
Source: PLoS One. 2020 Sep 24;15(9):e0238289. doi: 10.1371/journal.pone.0238289 (PMC7513996; doi:10.1371/journal.pone.0238289)
Supplement: S1 File — (DOC) [file pone.0238289.s001.doc]

| Fig # | Hazard Ratio | 95% Confidence Interval | Statistical Method Used | P value | # Samples |
| --- | --- | --- | --- | --- | --- |
| Fig. 1 | Patient selection flowchart | | Nil | | |
| Fig. 2A | KM curve (without/with anesthesia exposure) | | Log rank test | p < 0.001 | 34,371 |
|  | Crude Hazard Ratio | |  |  |  |
| without anesthesia | 1.000 |  |  |  |  |
| with anesthesia | 1.376 | 1.149-1.624 |  |  |  |
| Fig. 2B | KM curve (anesthesia frequency) | | Log rank test | p < 0.001 | 34,371 |
|  | Crude Hazard Ratio | |  |  |  |
| without anesthesia | 1.000 |  |  |  |  |
| 1 anesthesia event | 1.245 | 1.015-1.587 |  |  |  |
| 2 anesthesia events | 1.501 | 1.203-1.911 |  |  |  |
| ≥ 3 anesthesia events | 1.694 | 1.428-2.086 |  |  |  |
| Fig. 2C | KM curve (total anesthesia duration) | | Log rank test | p < 0.001 | 34,371 |
|  | Crude Hazard Ratio | |  |  |  |
| without anesthesia | 1.000 |  |  |  |  |
| < 2 hours | 1.240 | 1.106-1.382 |  |  |  |
| 2-4 hours | 1.561 | 1.264-1.980 |  |  |  |
| > 4 hours | 2.334 | 1.975-2.737 |  |  |  |
| Fig. 3 | Dose-response 3D plots | | Cox regression |  | 34,371 |
|  | Adjusted Hazard Ratio | |  |  |  |
| 0 events | 1.000 |  |  |  |  |
| 1 event, < 2 hours | 1.725 | 1.434-1.986 |  |  |  |
| 1 event, 2-4 hours | 2.170 | 1.899-2.384 |  |  |  |
| 1 event, > 4 hours | 2.358 | 2.075-2.712 |  |  |  |
| 2 event, < 2 hours | 2.099 | 1.801-2.496 |  |  |  |
| 2 event, 2-4 hours | 2.324 | 2.047-2.683 |  |  |  |
| 2 event, > 4 hours | 2.503 | 2.288-2.890 |  |  |  |
| 3 event, < 2 hours | 2.316 | 2.001-2.675 |  |  |  |
| 3 event, 2-4 hours | 2.718 | 2.462-3.187 |  |  |  |
| 3 event, > 4 hours | 2.992 | 2.599-3.506 |  |  |  |

Summary of figures and the statistical methods used
